# Minerals in the gut: scoping a Cambrian digestive system

**DOI:** 10.1098/rsos.160420

**Published:** 2016-11-16

**Authors:** K. M. Strang, H. A. Armstrong, D. A. T. Harper

**Affiliations:** Palaeoecosystems Group, Department of Earth Sciences, Durham University, Durham DH1 1LE, UK

**Keywords:** arthropod digestive system, taphonomy, Cambrian Lagerstätten

## Abstract

The Sirius Passet Lagerstätte of North Greenland contains the first exceptionally preserved mat-ground community of the Cambrian, dominated, in terms of abundance, by trilobites but particularly characterized by iconic arthropods and lobopods, some also occurring in the Burgess shale. High-resolution photography, scanning electron imaging and elemental mapping have been carried out on a variety of specimens of the non-mineralized arthropod *Campanamuta mantonae* (Budd 2011 *J. Syst. Palaeontol.* **9**, 217–260 (doi:10.1080/14772019.2010.492644)) which has three-dimensional gut and muscle preservation. Results show that the guts contain a high concentration of calcium phosphate (approximating to the mineral francolite), whereas the adjacent muscles are silicified. This indicates a unique, tissue-specific taphonomy for this Cambrian taxon. We hypothesize that the precipitation of calcium phosphate in the guts occurs rapidly after death by ‘crystal seed’ processes in suboxic, slightly acidic conditions; critically, the gut wall remained intact during precipitation. We postulate that the calcium phosphate was derived from ingested cellular material. Silicification of the muscles followed as the localized water chemistry became saturated in silica, high in Fe^2+^, and low in oxygen and sulfate. We document here the unique occurrence of two distinct but mechanistically similar taphonomic pathways within a diverse suite of possibilities in an Early Cambrian Lagerstätte.

## 1. Introduction

The fossil record offers us invaluable insights into important intervals in the history of life. The record is however generally biased by poor preservation, and even in exceptionally preserved specimens from fossil Lagerstätten, there is potential for key traits to be removed by decay. Sites that can be demonstrated to have very early, soft tissue preservation not only provide insights into taphonomic processes, but are also critical for reconstructing ancient communities and phylogenies [1]. This becomes particularly important when considering the very earliest radiations of bilaterian animals during the Cambrian explosion and hypotheses of likely ancestors in the Ediacara biota. A list of elements of the Sirius Passet (SP) fauna is provided by Peel & Ineson [2].

The Lower Cambrian black shales of SP, North Greenland preserve the oldest known examples of soft-bodied fossils from the Cambrian explosion together with more typical Cambrian skeletal animals. These include trilobites, other arthropods, lobopods, halkieriids and sponges [3]. The fauna is broadly similar to that found in the younger Burgess shale [4]. SP is located in J. P. Koch Fjord, N. Greenland, at 82°47.6′ N, 42°13.7′ W and during the Cambrian lay at approximately 10° S [2]. The locality was included in the ‘transitional’ Buen, and the fauna represents the earliest Cambrian community with exceptional preservation together with evidence for microbial mats, predating the Burgess shale by 10 Myr [2]. *Buenellus higginsi* is the most common macrofossil, indicative of the Laurentian Atdabanian/Botomian boundary, at the top of stage 3 to the base of stage 4, occurring between 511 and 521 Myr. However, precise correlation of the unit remains a subject of debate. The detailed petrography, mineralogy and metamorphic history were described by Strang *et al.* [5], and these properties are only outlined here. The shales are a poorly sorted mix largely consisting of quartz (60–65%), clay minerals, chloritoid porphyroblasts (indicating low P/T greenschist facies metamorphism) and silicified microbial mat material. A detailed description of the depositional environment, dating and metamorphic history has recently been presented [5].

The SP exhibits a diversity of taphonomic pathways; for example, the trilobites from the SP are preserved as complete, concave hyporelief external moulds and convex epirelief casts. External moulds are shown to consist of a thin veneer of authigenic silica. The casts are composed of silicified cyanobacterial mat material. Early silicification is supported by the presence of synsedimentary mat rip-up clasts, three-dimensional preservation, which indicates silicification prior to sediment compaction and textural and mono-CL evidence. The growth of metamorphic chloritoid needles, which crosscut the silica in the matrix, indicates that metamorphism occurred much later. Silicification was initiated by falling pH in the decaying mat. Pore waters are interpreted to have been initially alkali, silica-saturated, high in ferric iron but low in oxygen and sulfate. Excess silica was likely derived from remobilized biogenic silica, probably sponge spicules in the muddy sediment. It is not clear whether silicification was microbially or chemically mediated. The presence of cyanobacterial mats, sealing both the sediments and fossils was however fundamental to the preservation of this community.

*Campanamuta mantonae* [6] is also a common arthropod in the SP fauna and is non-mineralized. Approximately 1700 specimens have been reported to date [6]. Specimens are flattened but preserve three-dimensional axial traces, including gut tracts, diverticulae and muscle tissue [6]. The parts and counterparts often show different anatomical details in the axial region and sometimes exhibit cavities [6]. Distinguishing whether these cavities are the original anatomical features of the organism or are voids left by the later decay of soft parts or minerals is hard to determine. Budd [6] argued that the fossils are mostly (or completely) replacements of the original tissues, rather than preserved as moulds [6]. In this paper, we describe the taphonomy of these specimens and propose a taphonomic model for elements of the SP that enhances in some respects closer comparison with Lagerstätten from the Neoproterozoic than those from the Late Cambrian. The preservation of gut contents also allows for an interpretation of the mode of life of *C. mantonae*.

## 2. Material and methods

### 2.1. Specimens

**Figure 1.**
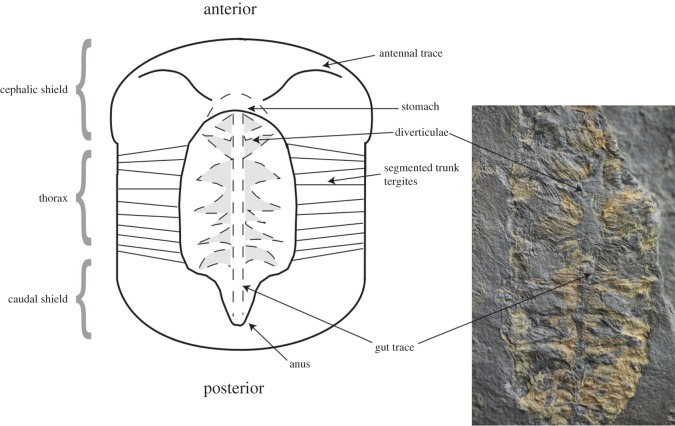
Diagram shows the main features, appendages and internal anatomy of *Campanamuta mantonae.* Photo inlay is a specimen of *C. mantonae* (MGUH 31567) that shows the gut tract and diverticulae which are most readily preserved. Specimen is 2 cm wide.

The numbered specimens (MGUH 31567–31572) including thin sections are reposited in the Natural History Museum of Denmark (Geological Museum), University of Copenhagen. Budd [6] described the anatomy of *C. mantonae* in considerable detail. It is a relatively large arthropod (mean length approx. 65 mm and width 35 mm) comprising three segments, with a smooth exoskeleton and a semicircular cephalic shield. The main morphological features are illustrated in figure 1. The external morphology is not well preserved. The only preserved internal anatomy is situated in the axial region and consists of the main digestive structures (figure 1). In rare specimens, the outline of the stomach can be identified, situated anteriorly in the cephalon. The gut tract extends posteriorly where it terminates at the anus. The triangular diverticulae are paired not only on either side of the gut, but also extend down towards the anus (figure 1).

**Figure 2.**
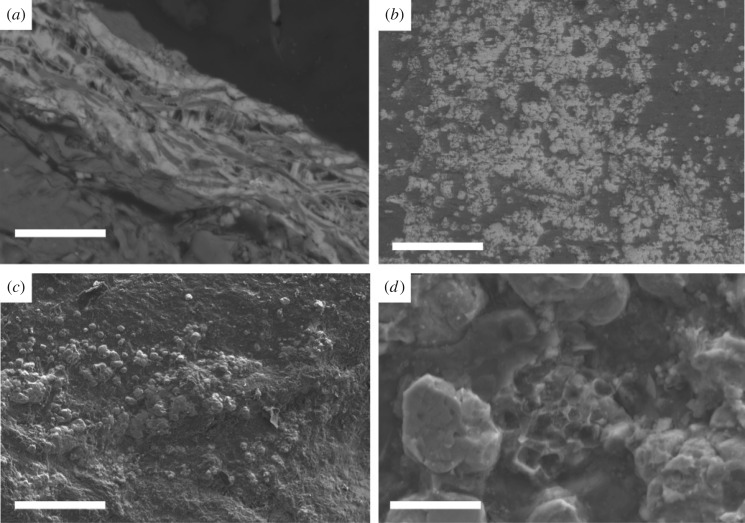
SEM and BSE images of the phosphate contained in the gut of *C. mantonae.* (*a*) SEM image of a cross section taken through a sample of *C. mantonae* (MGUH 31568), light grey material is phosphatized. Small pyrite framboids can be seen. Scale bar, 50 µm. (*b*) BSE image showing spherical texture of phosphate nodules in sample MGUH 31569. Scale bar, 100 µm. (*c*) Higher magnification SEM image shows spherical phosphate interpreted as possible microbial moulds. Scale bar, 20 µm (MGUH 31569). (*d*) High-resolution SEM image showing a pyrite framboid within the phosphate clusters. Scale bar, 10 µm (MGUH 31569).

### 2.2. Analysis

High-resolution photography was carried out on four samples of *C. mantonae* using a digital Cannon 5D mark III. Specimens were photographed, using low incidence lighting (directed from the NW). Contrast and saturation of the images were then then edited using Adobe Photoshop and Adobe Illustrator CS3 to reveal the best outlines of the preserved anatomy. Line drawings were constructed from photographs and camera lucida images. Specimens were carbon coated prior to scanning (approx. 20 µm thickness). This was undertaken, using the Hitachi SU-70 FEG SEM Facility (Department of Physics, Durham University). The machine was operated at 12 kV and with a current density of 46 µA. SEM-EDAX analysis was carried out using the backscatter detector and the same voltage settings used for imaging. For point analysis, a cobalt standard was run before analysis to provide quantitative results, analysed using QUANT software and running the standard several times to ensure maximum accuracy (100 ± 5%). SEM-cathodoluminescence (CL) was carried out using a mirror-type detector (Gatan Mono-CL). The machine was set to low magnification with a 10 kV and CL luminosities collected, using each colour filter (red > 600 nm, green > 480–580 nm and blue < 480 nm) and a panchromatic lens. Quartz grains display a variety of luminescence intensity dependent on their provenance. The standards used were adapted from Seyedolali *et al*. [7]. CL intensity is dependent on the density of intrinsic and extrinsic defects within the band gap of the mineral. These defects are usually structural imperfections in the quartz crystal owing to vacancies within the crystal lattice and can provide information on the conditions during mineralization and subsequent post-mineralization events such as deformation and metamorphism [8].

## 3. Results

**Figure 3.**
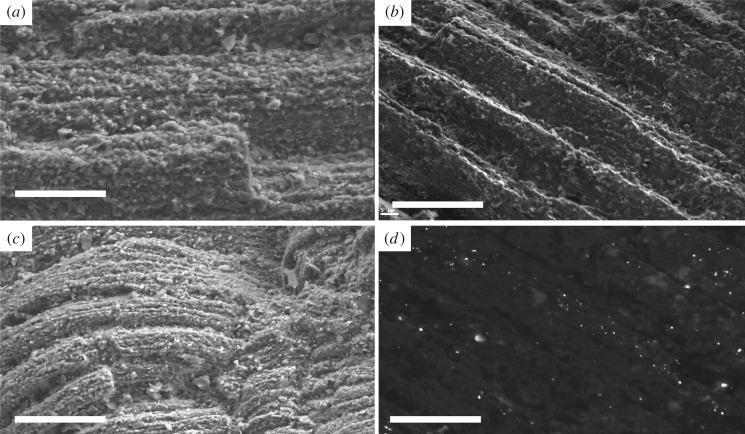
SEM, BSE and SEM-CL images of the silicified muscle tissue. (*a*) BSE image shows silicified muscle tissue. Scale bar, 20 µm (MGUH 31568). (*b*) SEM image of muscle tissue shows fibrous nature and small spherical nodules of silica. Scale bar, 20 µm (MGUH 31570). (*c*) BSE image showing truncated silicified muscle tissue. Scale bar, 20 µm (MGUH 31571). (*d*) SEM-CL image showing low monotone grey luminosity of the silica in the muscles, indicating similar formation conditions as that found in *Buenellus* [5] (MGUH 31572).

In specimens with digestive structures preserved, usually there is only limited preservation of the outer appendages. The area of the specimens outside the axial region is visible as a thin, dark film of silica. When preserved, bundles of muscle fibres adjacent to the digestive tract are yellow in colour and have three-dimensional relief. The muscle fibres track transversely and outwards from beside the diverticulae towards the thoracic tergites (figure 1) and exhibit well-preserved, oblique, micrometre-scale striations (figure 3*a*).

The gut of *C. mantonae* is a broad tube-like structure, which runs down the central axis, terminating at the anus (see also [6, fig. 1]). In the cephalic region (figure 1), the digestive tract consists of a sclerotized oesophagus which leads into the stomach, situated behind the head. The diverticulae are segmented and paired and open out from the gut [6, fig. 1]. The anus is clearly defined by a ring of plates [6, fig. 5*a*,*b*].

Three-dimensional gut traces contain high concentrations of calcium phosphate (Ca_3_(PO_4_)_2_; table 1) approximating to the mineral francolite. There are some traces of Si, Al and Fe but these are very minor and probably derived from the matrix. Phosphatization extends along the entire gut tract to the anus. The phosphatized areas have a sponge-like texture composed of small (less than 5 µm in diameter) spheres (figure 2*b*,*c*) organized into layers (figure 2*a*). The layers commonly contain small (less than 10 µm) pyrite framboids. There is no visible evidence for preserved biological material or sediment grains. In thin section, the boundary between the gut trace and the sediment below is sharp; there is no evidence in either hand specimen or thin section of preserved cuticle.

**Table 1.** EDAX data for phosphatized regions in sample SP0511. Data given in %.

spectrum	Al	Si	P	Ca	Fe	O
spectrum 1	1.90	0.00	21.34	20.04	0.00	56.36
spectrum 2	0.00	5.96	23.09	16.91	1.30	52.74
spectrum 3	0.67	1.42	19.67	36.18	0.00	42.06
spectrum 4	1.74	3.94	31.17	17.19	5.28	40.67

EDAX data confirm that muscle fibres are composed of finely microcrystalline silica (table 2) with very minute traces of Mg, possibly derived from the surrounding matrix. The preservation of these is relatively unvaried and forms distinct aligned blocks of muscle fibres, arranged *in situ*. The outer surfaces have a spherulitic texture at the micrometre scale (figure 3*a*). An approximately 5 µm thick layer of silica occurs at the edges of the phosphatized region (figure 2*a*). SEM-CL indicates the silica has very low luminosity with no distinct colour under both monochromatic filter and RGB filters (figure 3*d*).

**Table 2.** EDAX data for silicified regions in sample SP0511. Data given in %.

spectrum	Mg	Si	O
spectrum 1	3.09	43.65	53.26
spectrum 2	2.30	46.74	53.26
spectrum 3	2.28	44.98	52.74
spectrum 4	1.37	45.68	52.95

## 4. Discussion

The key drivers of decay are autolysis and microbial activity, and the latter is a key mediator in autogenic mineralization of soft tissues [9]. Microbial communities have the highest surface area to volume ratio of any group of living organisms and this combined with the abundance of charged chemicals and molecules on their surface makes them ideal environments for mineral nucleation and precipitation [10]. The gut traces in *C. mantonae* are preserved almost exclusively as francolite. The preservation of the three-dimensional structure indicates early mineralization prior to the compaction/collapse of the gut. Three-dimensional preservation of the gut is also commonly observed in a variety of taxa from Cambrian (and younger) Lagerstätten, indicating the gut is the most readily preserved internal structure. Examples include the Burgess shale arthropod *Leanchoilia, Odaria, Canadaspis, Perspicaris, Sidneyia, Anomalocaris* and *Opabinia* which all possess phosphatized midgut structures in their axial region [11,12]. *Myoscolex*, an Early Cambrian arthropod from the Emu Bay shale, is described as having only its trunk muscles phosphatized, which is in stark contrast to the preservation in the SP [13]. Phosphatized muscles are also found associated with hard parts in the Mesozoic, such as the muscle tissue in the horseshoe crab *Mesolimulus* [14] and preservation of muscles in phosphate from the Konservat-Lagerstätten in Lebanon [15].

The close proximity of distinct tissues with distinct taphonomies is extremely unusual. Experimental decay studies of modern brine shrimp *Artemia* indicate chemistry of the gut contents and the presence of endogenous bacteria are the key factors in creating a unique microenvironment in which the gut is preserved [9]. The nature of the preservation of the surrounding tissues is dependent on whether the gut wall remains intact. If the gut ruptures, the endogenous microbes leak into the body cavity where they control the tissue preservation through mineral templating [9]. In the *C. mantonae* specimens from the SP, only the gut is phosphatized, and the remaining axial tissues and structures are preserved as silica replacement. This would indicate the gut wall remained intact and that endogenous bacteria remained within the gut and were responsible for the preservation of the gut.

Phosphatization and silicification occur under markedly different environmental and chemical conditions [16]. Phosphatization is widely recognized in the preservation of soft tissues [10,17]. There are two sources of phosphate: (i) from the breakdown of organic matter during bacterial sulfate reduction and (ii) phosphate can also be released from absorption sites on ferric oxyhydroxide, during reduction of the iron (Fe^3+^ > Fe^2+^ [18]). Phosphatization occurs predominantly in a suboxic environment within the upper few centimetres of the sediment [19], at lowered pH induced by decaying organic matter and with sufficient time under these conditions, free from scavengers [17,20].

Decay experiments have shown that the digestive and other internal organs of marine arthropods are particularly prone to rapid decay (2–3 days) and liquidation under open, aerobic conditions at room temperature [9,21]. Butler *et al*. [9] also showed that the carcass of the brine shrimp was rapidly consumed (2–3 days) by endogeneous, gut-derived microbes and pervasive phosphatization of the internal tissues occurred following the rupture of the gut wall. Decay rarely lasts longer than one month [22]. By inference, the three-dimensional preservation of the guts in the *C. mantonae* specimens must have started very early after the death of the organism when the gut wall remained. The precipitation of calcium phosphate is favoured over calcium carbonate under slightly acidic marine conditions [20].

Both sulfide-oxidizing bacteria (SOB) and sulfate-reducing bacteria (SRB) have been implicated in the precipitation of phosphate in the marine environment [13,14,23,24]. SOB are able to store polyphosphate under oxic conditions, this polyphosphate being used as an additional energy source [25]. Examples of SOB include *Thioploca, Beggiatoa* and *Thiomargarita,* and these taxa are major components in the benthic sulfur cycle, where they reoxidize sulfide ([26] and references therein). SRB facilitate phosphate precipitation by increasing the phosphate concentrations in pore waters through the decay of organic matter. Degradation of organic matter by SRB is a predominantly anaerobic process, where supersaturation with respect to phosphate is commonly reached [26]. The presence of pyrite framboids within the phosphate supports the role of SRB in the release of PO_4_^−^ from the organic gut contents, from either ingested seawater or iron-rich sediment (see also [11]). We have no evidence from imaging for the presence of sediment particles within the gut material and conclude the phosphate was derived from ingested organic material. Furthermore, the absence of phosphate in the rock matrix also suggests it came from an internal source.

The molecular initiation and aggregation of silica plays a major role in biosilicification and the presence of proteinaceous material resulting from decay [27]. In microbially mediated silica precipitation, it has been shown that microbial surfaces do not directly nucleate silica mineral formation; however, they play an important role in the aggregation of polymeric silica and the deposition of silica colloids on microbial surfaces, for example in modern hot spring environments, silica sinters actively form in close spatial relation to microorganisms ([28] and references within). In the former, direct precipitation of silica into void spaces is the likely scenario in the SP [5]. Sedimentary factors that control silicification are the permeability of sediment, silica availability (both in the pore waters and sediment) and the concentration of organic matter ([29] and references therein; [30]). Silica-rich pore waters were likely derived from remobilization of biogenic silica from sponge spicules [5]. Soluble silica, in the form of monosilicic acid, H_4_SiO_4_, dissociates to H_3_SiO_4_^–^ at pH values above *ca* 9.7 [31,32]. H_3_SiO^–^ is a highly soluble form of silicic acid, and it reacts with hydrogen ions to form SiO_2_ [33, fig. 3].

Silica precipitation is sensitive to the iron content of bottom/pore waters and in Early Cambrian seawater and Fe^2+^ appears to be high relative to FeOOH [16]. Unlike the trilobite specimens, silicification in the non-mineralized *C. mantonae* is restricted to the muscles. Muscle tissue in modern arthropods is composed of actin and myosin [34] and it is likely that muscle tissue in extinct arthropods had a similar composition. While the involvement of microbes in silica precipitation cannot be directly excluded, more extensive mineralization of all the tissues might be expected. Alternatively, the arrangement of muscles into micrometre-scale fibres provides an excellent substrate for silica precipitation, as it creates a large surface area comprising the reactive proteinaceous and amino acids necessary for initial silica aggregation [29]. Three-dimensionally preserved internal organs have also been documented from the Burgess shale in the Stephen formation (Cambrian) [11] annelids from the Cretaceous Konservat-Lagerstätten of Hakel and Hjoula, Lebanon [15], the Guizhou Province in South China (see [12,35]) and are also associated with flattened hard parts, interpreted as a result of syndiagenetic microbial decay.

## 5. Taphonomic pathway

**Figure 4.**
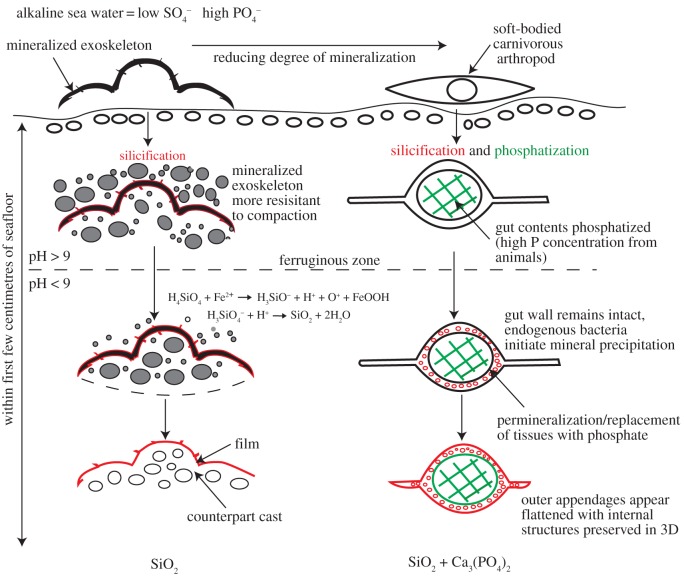
Schematic model shows the preferred taphonomic pathway of non-mineralizing *C. mantonae* (right) compared with that of mineralizing *Buenellus* (left).

Figure 4 shows the proposed taphonomic pathway and compares this to that proposed for the mineralized trilobite *Buenellus*. The precipitation of two distinct mineral phases in the gut and axial muscle fibres of *C. mantonae* indicates the formation in different and isolated microenvironments along a redox gradient. Phosphatization of the gut contents occurred in slightly acidic, suboxic conditions, under the control of endogenous SRB, within days of death and therefore at the seafloor. The three-dimensional preservation indicates of the gut trace was fully permineralized before specimen collapse during decay, burial and sedimentary compaction. Silicification of the adjacent muscle fibres occurred in the presence of silica-saturated pore waters with a pH around 9.7 within the microbial mat.

The relative timing of these two processes is not easily constrained. That both tissues are preserved in three dimensions suggests both were mineralized early during decay. Postmortem sealing of the specimens by cyanobacterial mats has been hypothesized as a mechanism for rapid sealing of the specimens from decay and predation, resulting in a localized pH environment suitable for controlling the silicification of the mineralized trilobites in the SP [5]. It is possible that silicification was initiated once the specimens became entombed in the decaying microbial mat and this would have provided the necessary conditions for decay-related reduction in pH > 7 [5,31].

## 6. Gut morphology and ecological implications

Well-preserved digestive systems in Cambrian arthropods display a variety of biserial midgut glands, from simple bunch-like or lobe-like digestive glands to complex branching features [36]. This suggests that, like their modern relatives, these arthropods ingested phosphate from their food sources [36]. *C. mantonea* possessed a relatively complex gut consisting of a tube-like structure which runs down the central axis of the animal (figure 1) [6, fig. 5*a*,*b*]. Paired leaf-like diverticulae are attached to the midgut and provide evidence that *C. mantonea* would have been able to process and digest more complex food sources, such as smaller arthropods. In living branchiurans, the diverticulae are used to store food prior to enzymatic breakdown and the absorption of nutrients in a rich but infrequent diet [11,36]. The evolution of more complex guts with digestive glands work by allowing the animal to increase the efficiency of food processing by increasing the surface area between nutrients and epithelial tissues, thus allowing larger particles to be consumed which, in turn, would have enabled them to uphold the energy demands of a more active and predatory lifestyle [36]. The ability to process larger food particles would have been clearly advantageous for active Cambrian arthropods. Unlike their modern counterparts, early Cambrian arthropods do not possess a large array of differentiated appendages used for capturing and breaking up prey [36]. *C. mantonae* only appears to have possessed long, slender antennae [6] (figure 1) which would have acquired a sensory function. The antennae only protruded a short distance from the anterior shield margin, as most of their length is hidden under the shield [6]. Protection by the shield may be the reason for antennae being the most readily preserved appendage of this animal. Evidence that other trilobites grazed the microbial mat [5] and the lack of sediment particles in the gut suggest that *C. mantonae* may have been consuming other smaller arthropods, which would have provided a rich source of phosphate. It would appear predators, in significant numbers, occupied both the benthos and nekton in the Early Cambrian.

## 7. Conclusion

Detailed micrometre-scale analysis of the distribution of phosphate and silica in relation to the internal morphological structure of the soft-bodied arthropod *C. mantonae* from SP indicates a complex tissue-specific preservation of internal structures. The guts contain a high concentration of calcium phosphate (approximating to francolite), whereas the adjacent muscles are silicified_._ We hypothesize that the precipitation of calcium phosphate in the guts occurs rapidly after death by ‘crystal seed’ processes in suboxic, slightly acidic conditions; critically, the gut wall remained intact during precipitation. We postulate the calcium phosphate was derived from ingested organic material. Silicification of the muscles followed as the localized water chemistry became saturated in silica, high in Fe^2+^, and low in oxygen and sulfate. These modes of preservation indicate a diversity of taphonomic pathways in faunas, chronostratigraphically intermediate between the Neoproterozoic Ediacara biota and the Middle Cambrian Burgess shale. The absence of sediment particles in the gut suggests that *C. mantonae* was either a scavenger or predating smaller arthropods.

## Data accessibility

The numbered specimens (MGUH 31567–31572) are reposited in the Natural History Museum of Denmark (Geological Museum), University of Copenhagen.

## References

[1] Sansom RS, Gabbott SE, Purnell MA 2010 Non-random decay of chordate characters causes bias in fossil interpretation. Nature 463, 797–800. (doi:10.1038/nature08745) 20118914 10.1038/nature08745

[2] Peel JS, Ineson JR 2011 The extent of the Sirius Passet Lagerstätte (early Cambrian) of North Greenland. Bull. Geosci. 86, 535–543. (doi:10.3140/bull.geosci.1269)

[3] Morris SC, Peel JS 2008 The earliest annelids: lower Cambrian polychaetes from the Sirius Passet Lagerstätte, Peary Land, North Greenland. Acta Palaeontol. Pol. 53, 137–148. (doi:10.4202/app.2008.0110)

[4] Smith MP, Harper DAT 2013 Earth science. Causes of the Cambrian explosion. Science 341, 1355–1356. (doi:10.1126/science.1239450) 24052300 10.1126/science.1239450

[5] Strang KM, Armstrong HA, Harper DAT, Trabucho-Alexandre JP 2016 The Sirius Passet Lagerstätte: silica death masking opens the window on the earliest matground community of the Cambrian explosion. Lethaia 49, 631–643. (doi:10.1111/let.12174)

[6] Budd GE 2011 *Campanamuta mantonae* gen. et. sp. nov., an exceptionally preserved arthropod from the Sirius Passet Fauna (Buen Formation, lower Cambrian, North Greenland). J. Syst. Palaeontol. 9, 217–260. (doi:10.1080/14772019.2010.492644)

[7] Seyedolali A, Krinsley DH, Boggs S, O'Hara PF, Dypvik H, Goles GG 1997 Provenance interpretation of quartz by scanning electron microscope–cathodoluminescence fabric analysis. Geology 25, 787–790. (doi:10.1130/0091-7613(1997)025<0787:PIOQBS>2.3.CO;2)

[8] Frelinger SN, Ledvina MD, Kyle JR, Zhao D 2015 Scanning electron microscopy cathodoluminescence of quartz: principles, techniques and applications in ore geology. Ore Geol. Rev. 65, 840–852. (doi:10.1016/j.oregeorev.2014.10.008)

[9] Butler AD, Cunningham JA, Budd GE, Donoghue PCJ 2015 Experimental taphonomy of Artemia reveals the role of endogenous microbes in mediating decay and fossilization. Proc. R. Soc. B 282, 20150476 (doi:10.1098/rspb.2015.0476) 10.1098/rspb.2015.0476 PMC4455810 25972468

[10] Douglas S, Beveridge TJ 1998 Mineral formation by bacteria in natural microbial communities. FEMS Microbiol. Ecol. 26, 79–88. (doi:10.1111/j.1574-6941.1998.tb00494.x)

[11] Butterfield NJ 2002 Leanchoilia guts and the interpretation of three-dimensional structures in Burgess Shale-type fossils. Paleobiology 28, 155–171. (doi:10.1666/0094-8373(2002)028<0155:LGATIO>2.0.CO;2)

[12] Lin J-P, Briggs DEG 2010 Burgess shale-type preservation: a comparison of Naraoiids (Arthropoda) from three Cambrian localities. Palaios 25, 463–467. (doi:10.2110/palo.2009.p09-145r)

[13] Briggs DEG, Nedin C. 1997 The taphonomy and affinities of the problematic fossil *Myoscolex* from the Lower Cambrian Emu Bay shale of South Australia. J. Paleontol. 71, 22–32. (doi:10.1017/S0022336000038919)

[14] Briggs DE, Moore RA, Shultz JW, Schweigert G 2005 Mineralization of soft-part anatomy and invading microbes in the horseshoe crab *Mesolimulus* from the Upper Jurassic Lagerstätte of Nusplingen, Germany. Proc. R. Soc. B 272, 627–632. (doi:10.1098/rspb.2004.3006) 10.1098/rspb.2004.3006 PMC1564078 15817437

[15] Wilson P, Parry LA, Vinther J, Edgecombe GD 2016 Unveiling biases in soft-tissue phosphatization: extensive preservation of musculature in the Cretaceous (Cenomanian) polychaete *Rollinschaeta myoplena* (Annelida: Amphinomidae). Palaeontology 59, 463–479. (doi:10.1111/pala.12237)

[16] Muscente AD, Hawkins AD, Xiao S 2014 Fossil preservation through phosphatization and silicification in the Ediacaran Doushantuo formation (South China): a comparative synthesis. Palaeogeogr. Palaeoclimatol. Palaeoecol. 434, 46–62. (doi:10.1016/j.palaeo.2014.10.013)

[17] Wilby PR, Briggs DEG, Bernier P, Gaillard C 1996 Role of microbial mats in the fossilization of soft tissues. Geology 24, 787–790. (doi:10.1130/0091-7613(1996)024<0787:ROMMIT>2.3.CO;2)

[18] Krom MD, Berner RA 1980 Adsorption of phosphate in anoxic marine sediments. Limnol. Oceanogr. 25, 797–806. (doi:10.4319/lo.1980.25.5.0797)

[19] Jahnke RA, Emerson SR, Roe KK, Burnett WC 1983 The present day formation of apatite in Mexican continental margin sediments. Geochim. Cosmochim. Acta 47, 259–266. (doi:10.1016/0016-7037(83)90138-2)

[20] Lerosey-Aubril R, Hegna TA, Kier C, Bonino E, Habersetzer J, Carré M 2012 Controls on gut phosphatisation: the trilobites from the Weeks Formation Lagerstätte (Cambrian; Utah). PLoS ONE 7, e32934 (doi:10.1371/journal.pone.0032934) 22431989 10.1371/journal.pone.0032934 PMC3303877

[21] Hof CHJ, Briggs DEG 1997 Decay and mineralization of mantis shrimps (Stomatopoda; Crustacea); a key to their fossil record. Palaios 12, 420–438. (doi:10.1043/0883-1351(1997)012)

[22] Fatka O, Budil P, David M 2015 Digestive structures in Ordovician trilobites *Colpocoryphe* and *Flexicalymene* from the Barrandian area of Czech Republic. Est. J. Earth Sci. 64, 255–266. (doi:10.3176/earth.2015.32)

[23] Briggs DEG 2003 The role of decay and mineralization in the preservation of soft-bodied fossils. Annu. Rev. Earth Planet. Sci. 31, 275–301. (doi:10.1146/annurev.earth.31.100901.144746)

[24] Wilby PR, Briggs DEG 1997 Taxonomic trends in the resolution of detail preserved in fossil phosphatized soft tissues. Geobios 30, 493–502. (doi:10.1016/S0016-6995(97)80056-3)

[25] Schulz HN, Schulz HD 2005 Large sulfur bacteria and the formation of phosphorite. Science 307, 416–418. (doi:10.1126/science.1103096) 15662012 10.1126/science.1103096

[26] Arning ET, Birgel D, Brunner B, Peckmann J 2009 Bacterial formation of phosphatic laminites off Peru. Geobiology 7, 295–307. (doi:10.1111/j.1472-4669.2009.00197.x) 19476504 10.1111/j.1472-4669.2009.00197.x

[27] Belton D, Paine G, Patwardhan SV, Perry CC 2004 Towards an understanding of (bio)silicification: the role of amino acids and lysine oligomers in silicification. J. Mater. Chem. 14, 2231–2241. (doi:10.1039/b401882f)

[28] Yee N, Phoenix VR, Konhauser KO, Benning LG, Ferris FG 2003 The effect of cyanobacteria on silica precipitation at neutral pH: implications for bacterial silicification in geothermal hot springs. Chem. Geol. 199, 83–90. (doi:10.1016/S0009-2541(03)00120-7)

[29] Butts SH 2014 Silicification. In *Reading and writing of the fossil record: preservational pathways to exceptional fossilization* (eds M Laflamme, JD Schiffbauer, SAF Darroch). The Paleontological Society Papers, vol. 20, pp. 15–33.

[30] Akahane H, Furuno T, Miyajima H, Yoshikawa T, Yamamoto S 2004 Rapid wood silicification in hot spring water: an explanation of silicification of wood during the Earth's history. Sediment. Geol. 169, 219–228. (doi:10.1016/j.sedgeo.2004.06.003)

[31] Birnbaum J, Wireman JW 1985 Sulfate-reducing bacteria and silica solubility: a possible mechanism for evaporite diagenesis and silica precipitation in banded iron formations. Can. J. Earth Sci. 22, 1904–1909. (doi:10.1139/e85-206)

[32] Arp G, Reimer A, Reitner J 2003 Microbialite formation in seawater of increased alkalinity, Satonda Crater Lake, Indonesia. J. Sediment. Res. 73, 105–127. (doi:10.1306/071002730105)

[33] Rickert D, Schlüter M, Wallmann K 2002 Dissolution kinetics of biogenic silica from the water column to the sediments. Geochim. Cosmochim. Acta 66, 439–455. (doi:10.1016/S0016-7037(01)00757-8)

[34] Neville AC 2012 Biology of the arthropod cuticle. Berlin, Germany: Springer Science, Business Media.

[35] Lin J-P. 2006 Taphonomy of Naraoiids (Arthropoda) from the Middle Cambrian Kaili Biota, Guizhou Province, South China. Palaios 21, 15–25. (doi:10.2110/palo.2004.p04-83)

[36] Vannier J, Liu J, Lerosey-Aubril R, Vinther J, Daley AC 2014 Sophisticated digestive systems in early arthropods. Nat. Commun. 5, 3641 (doi:10.1038/ncomms4641) 24785191 10.1038/ncomms4641

